# 2-Hy­droxy-5-[(*E*)-2-methyl­benzyl­idene]-8-(2-methyl­phen­yl)-9-phenyl-3,10-diaza­hexa­cyclo­[10.7.1.1^3,7^.0^2,11^.0^7,11^.0^16,20^]henicosa-1(20),12,14,16,18-pentaen-6-one

**DOI:** 10.1107/S160053681204648X

**Published:** 2012-11-17

**Authors:** Abdulrahman I. Almansour, Raju Suresh Kumar, Natarajan Arumugam, Hasnah Osman, J. Suresh

**Affiliations:** a Department of Chemistry, College of Sciences, King Saud University, PO Box 2455, Riyadh 11451, Saudi Arabia; b School of Chemical Sciences, Universiti Sains Malaysia, 11800 USM, Penang, Malaysia; c Department of Physics, The Madura College, Madurai 625 011, India

## Abstract

In the title compound, C_40_H_34_N_2_O_2_, the central piperidine ring adopts a half-chair conformation and the fused pyrrolidine rings adopt twisted envelope (with the C atom bearing the methylphenyl ring as the flap atom) and envelope (with the C atom bound to the N atom, common to the pyridinone and pyrrolidine rings being the flap atom) conformations. The mol­ecular structure features weak intra­molecular N—H⋯O and C—H⋯O inter­actions. In the crystal, O—H⋯O hydrogen bonds generate a *C*(7) chain along the *b-*axis direction. C—H⋯O inter­actions also occur.

## Related literature

For hydrogen-bond motifs, see: Bernstein *et al.* (1995 ▶). For similar structures, see: Kumar *et al.* (2010 ▶, 2011 ▶, 2012 ▶). For the importance of pyrrolidine, see: Asano *et al.* (2000 ▶); Shorvon (2001 ▶); Watson *et al.* (2001 ▶); Winchester & Fleet (1992 ▶). For puckering parameters, see: Cremer & Pople (1975 ▶).
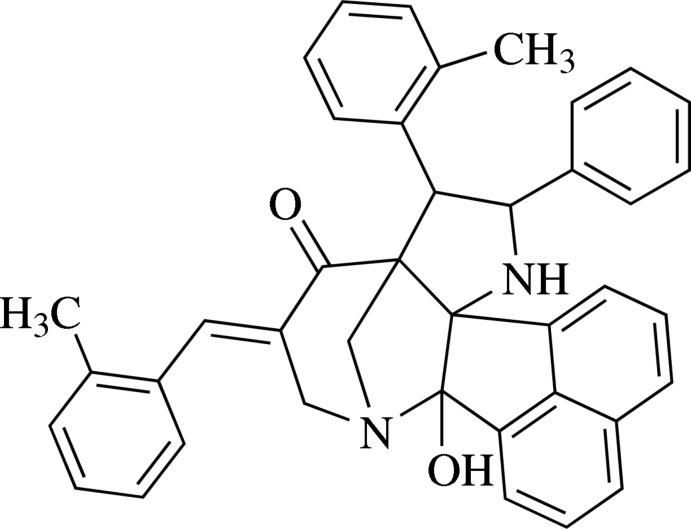


## Experimental

### 

#### Crystal data

C_40_H_34_N_2_O_2_
*M* *_r_* = 574.69
Monoclinic, 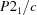

*a* = 14.0679 (2) Å
*b* = 7.7245 (1) Å
*c* = 26.9686 (3) Å
β = 92.596 (1)°
*V* = 2927.60 (7) Å^3^
*Z* = 4
Mo *K*α radiation
μ = 0.08 mm^−1^
*T* = 293 K
0.21 × 0.15 × 0.13 mm

#### Data collection

Bruker Kappa APEXII diffractometer
Absorption correction: multi-scan (*SADABS*; Sheldrick, 1996 ▶) *T*_min_ = 0.973, *T*_max_ = 0.978
33520 measured reflections
8777 independent reflections
6575 reflections with *I* > 2σ(*I*)
*R*_int_ = 0.047

#### Refinement

*R*[*F* ^2^ > 2σ(*F* ^2^)] = 0.054
*wR*(*F* ^2^) = 0.137
*S* = 1.02
8777 reflections
404 parameters
H atoms treated by a mixture of independent and constrained refinement
Δρ_max_ = 0.48 e Å^−3^
Δρ_min_ = −0.27 e Å^−3^

### 

Data collection: *APEX2* (Bruker, 2004 ▶); cell refinement: *SAINT* (Bruker, 2004 ▶); data reduction: *SAINT*; program(s) used to solve structure: *SHELXS97* (Sheldrick, 2008 ▶); program(s) used to refine structure: *SHELXL97* (Sheldrick, 2008 ▶); molecular graphics: *PLATON* (Spek, 2009 ▶); software used to prepare material for publication: *SHELXL97*.

## Supplementary Material

Crystal structure: contains datablock(s) global, I. DOI: 10.1107/S160053681204648X/pk2453sup1.cif

Structure factors: contains datablock(s) I. DOI: 10.1107/S160053681204648X/pk2453Isup2.hkl

Supplementary material file. DOI: 10.1107/S160053681204648X/pk2453Isup3.cml

Additional supplementary materials:  crystallographic information; 3D view; checkCIF report

## Figures and Tables

**Table 1:** Hydrogen-bond geometry (Å, °)

*D*—H⋯*A*	*D*—H	H⋯*A*	*D*⋯*A*	*D*—H⋯*A*
O2—H2⋯O1^i^	0.82	2.02	2.7828 (15)	155
C1—H1*A*⋯O2^ii^	0.97	2.46	3.3040 (16)	145
C57—H57*B*⋯O1	0.96	2.59	3.3859 (18)	141
N2—H2*A*⋯O2	0.92 (2)	2.27 (2)	2.8016 (18)	117 (2)

Symmetry codes: (i) 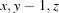; (ii) 
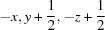
.

**Fig. 1.**
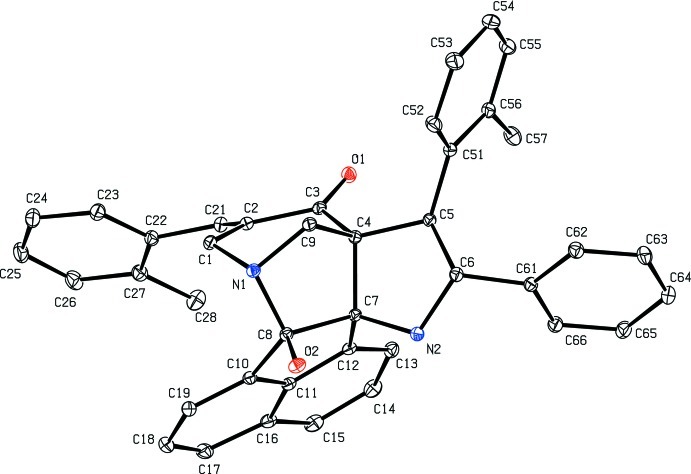
The molecular structure of (I), showing 50% probability displacement ellipsoids and the atom-numbering scheme.

**Fig. 2.**
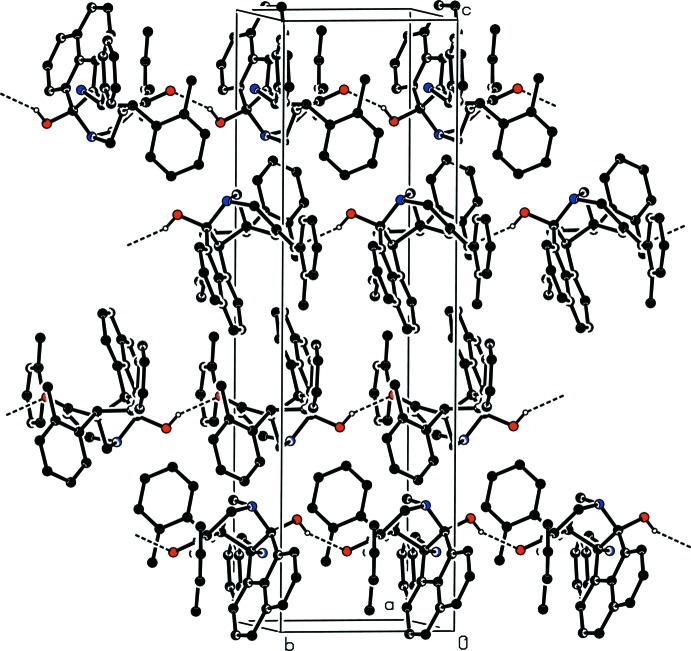
A packing diagram viewed roughly down the a-axis, showing the H-bond motif C11(7).

## supplementary crystallographic information

### Comment

The pyrrolidine ring system has been the subject of research for more than three decades. Many natural and synthetic compounds with pyrrolidine moieties have received much attention because of their remarkable biological properties (Shorvon, 2001; Watson *et al.*, 2001; Asano *et al.*, 2000; Winchester *et al.*, 1992). Recognizing the importance of such compounds in drug discovery and as a part of our ongoing research in the construction of novel heterocycles, has prompted us to investigate the 1,3-dipolar cycloaddition of bisarylmethylidene pyridinones with azomethine ylide generated *in situ* from acenaphthenequinone and proline, we and report the crystal structure of the resulting pyrrolidine cyclo-adduct in this paper.

In the title compound,*C*_40_H_34_N_2_ O_2_, the piperidine ring (N1/C1—C4/C9) adopts a half-chair conformation [Q = 0.6188 (2) Å, θ = 142.52 (2)°, φ = 123.6 (2)°; Cremer & Pople, 1975] which is in close agreement with those of the other related structures (Kumar *et al.* 2010; Kumar *et al.*2011; Kumar *et al.* 2012). The two fused pyrrolidine rings with atom sequences (N1/C4/C7—C9) and (N2/C4—C7), adopt a twisted envelope conformation (C9 atom as the flap) and an envelope conformation (C5 atom as the flap) respectively. The puckering parameters are Q = 0.4648 (15) Å, φ =325.20 (19)° for the N1/C4/C7—C9) pyrrolidine ring and Q = 0.3918 (16) Å, φ = 77.9 (2)° for the (N2/C4—C7) pyrrolidine ring. In the structure, the aryl ring C22—C27 is not coplanar with the mean plane of the piperidone ring [torsion angle C1—C2—C21—C22 is 5.77 (3)] °, which is due to non-bonded interactions between one of the *ortho* H atoms in the aryl ring and the equatorial H atom at the 2-position of the piperidone ring (H12A/H1A or H1B).

The molecular structure features weak intra-molecular N—H···O and C—H···O interactions. Intermolecular O2—H2···O1 bonds form an infinite one-dimensional chain parallel to the *b* axis, in a C_1_^1^(7) motif (Bernstein *et al.*, 1995).

### Experimental

A mixture of 3,5-bis[(*E*)-(2-methylphenyl)methylidene]tetrahydro-4(1*H*)-pyridinone (1 mmol), acenaphthenequinone (1 mmol), and phenylglycine (1 mmol) were dissolved in methanol (5 ml) and refluxed in a water bath for 1 h. After completion of the reaction as evident from TLC, the mixture was poured into water (50 ml). The precipitated solid was filtered and washed with water to obtain the product which was further purified by recrystallization from ethyl acetate. Yield 89%, melting point 212–213°C

### Refinement

H atoms were placed at calculated positions and allowed to ride on their carrier atoms with C—H = 0.93–0.97 Å and O—H = 0.82 Å. *U*_iso_ = 1.2*U*_eq_(C) for CH CH_2_ groups and *U*_iso_ = 1.5*U*_eq_(C) for OH and CH_3_ groups.

### Crystal data

**Table:**

C_40_H_34_N_2_O_2_	*F*(000) = 1216
*M**_r_* = 574.69	*D*_x_ = 1.304 Mg m^−^^3^
Monoclinic, *P*2_1_/*c*	Mo *K*α radiation, λ = 0.71073 Å
Hall symbol: -P 2ybc	Cell parameters from 2000 reflections
*a* = 14.0679 (2) Å	θ = 2–31°
*b* = 7.7245 (1) Å	µ = 0.08 mm^−^^1^
*c* = 26.9686 (3) Å	*T* = 293 K
β = 92.596 (1)°	Block, colourless
*V* = 2927.60 (7) Å^3^	0.21 × 0.15 × 0.13 mm
*Z* = 4	

### Data collection

**Table:**

Bruker Kappa APEXII diffractometer	8777 independent reflections
Radiation source: fine-focus sealed tube	6575 reflections with *I* > 2σ(*I*)
Graphite monochromator	*R*_int_ = 0.047
Detector resolution: 0.2 pixels mm^-1^	θ_max_ = 30.4°, θ_min_ = 1.5°
ω and φ scans	*h* = −20→19
Absorption correction: multi-scan (*SADABS*; Sheldrick, 1996)	*k* = −10→10
*T*_min_ = 0.973, *T*_max_ = 0.978	*l* = −38→38
33520 measured reflections	

### Refinement

**Table:**

Refinement on *F*^2^	Primary atom site location: structure-invariant direct methods
Least-squares matrix: full	Secondary atom site location: difference Fourier map
*R*[*F*^2^ > 2σ(*F*^2^)] = 0.054	Hydrogen site location: inferred from neighbouring sites
*wR*(*F*^2^) = 0.137	H atoms treated by a mixture of independent and constrained refinement
*S* = 1.02	*w* = 1/[σ^2^(*F*_o_^2^) + (0.0605*P*)^2^ + 1.2845*P*] where *P* = (*F*_o_^2^ + 2*F*_c_^2^)/3
8777 reflections	(Δ/σ)_max_ < 0.001
404 parameters	Δρ_max_ = 0.48 e Å^−^^3^
0 restraints	Δρ_min_ = −0.27 e Å^−^^3^

### Special details

**Table:**

Geometry. All e.s.d.'s (except the e.s.d. in the dihedral angle between two l.s. planes) are estimated using the full covariance matrix. The cell e.s.d.'s are taken into account individually in the estimation of e.s.d.'s in distances, angles and torsion angles; correlations between e.s.d.'s in cell parameters are only used when they are defined by crystal symmetry. An approximate (isotropic) treatment of cell e.s.d.'s is used for estimating e.s.d.'s involving l.s. planes.
Refinement. Refinement of *F*^2^ against ALL reflections. The weighted *R*-factor *wR* and goodness of fit *S* are based on *F*^2^, conventional *R*-factors *R* are based on *F*, with *F* set to zero for negative *F*^2^. The threshold expression of *F*^2^ > σ(*F*^2^) is used only for calculating *R*-factors(gt) *etc*. and is not relevant to the choice of reflections for refinement. *R*-factors based on *F*^2^ are statistically about twice as large as those based on *F*, and *R*- factors based on ALL data will be even larger.

### Fractional atomic coordinates and isotropic or equivalent isotropic displacement parameters (Å^2^)

**Table:**

	*x*	*y*	*z*	*U*_iso_*/*U*_eq_	
H2A	0.1618 (13)	−0.048 (3)	0.1407 (7)	0.021 (5)*	
C1	−0.08779 (9)	0.3112 (2)	0.19923 (5)	0.0140 (3)	
H1A	−0.0948	0.3696	0.2307	0.017*	
H1B	−0.1485	0.2587	0.1897	0.017*	
C2	−0.06395 (9)	0.4456 (2)	0.16030 (5)	0.0138 (3)	
C3	0.03856 (9)	0.4691 (2)	0.14943 (4)	0.0130 (3)	
C4	0.10336 (9)	0.31845 (19)	0.16240 (5)	0.0120 (3)	
C5	0.20821 (9)	0.34675 (19)	0.15125 (5)	0.0123 (3)	
H5	0.2092	0.3925	0.1174	0.015*	
C6	0.24693 (9)	0.1597 (2)	0.14945 (5)	0.0136 (3)	
H6	0.2609	0.1174	0.1833	0.016*	
C7	0.07621 (9)	0.15392 (19)	0.13098 (5)	0.0116 (3)	
C8	−0.00249 (9)	0.06007 (19)	0.16306 (5)	0.0124 (3)	
C9	0.07764 (9)	0.2551 (2)	0.21505 (5)	0.0139 (3)	
H9A	0.1241	0.1725	0.2282	0.017*	
H9B	0.0740	0.3515	0.2379	0.017*	
C10	−0.08848 (9)	0.0412 (2)	0.12769 (5)	0.0135 (3)	
C11	−0.06725 (9)	0.1136 (2)	0.08159 (5)	0.0133 (3)	
C12	0.02524 (9)	0.18289 (19)	0.08079 (5)	0.0127 (3)	
C13	0.05395 (10)	0.2586 (2)	0.03803 (5)	0.0167 (3)	
H13	0.1141	0.3078	0.0367	0.020*	
C14	−0.01019 (11)	0.2604 (2)	−0.00437 (5)	0.0202 (3)	
H14	0.0096	0.3098	−0.0336	0.024*	
C15	−0.10010 (10)	0.1918 (2)	−0.00362 (5)	0.0196 (3)	
H15	−0.1397	0.1938	−0.0322	0.024*	
C16	−0.13281 (10)	0.1180 (2)	0.04057 (5)	0.0163 (3)	
C17	−0.22466 (10)	0.0493 (2)	0.04838 (5)	0.0201 (3)	
H17	−0.2712	0.0515	0.0228	0.024*	
C18	−0.24517 (10)	−0.0208 (2)	0.09376 (6)	0.0206 (3)	
H18	−0.3058	−0.0645	0.0981	0.025*	
C19	−0.17692 (10)	−0.0282 (2)	0.13393 (5)	0.0170 (3)	
H19	−0.1917	−0.0790	0.1639	0.020*	
C21	−0.12729 (9)	0.5353 (2)	0.13163 (5)	0.0152 (3)	
H21	−0.1016	0.6046	0.1074	0.018*	
C22	−0.23170 (9)	0.5386 (2)	0.13336 (5)	0.0159 (3)	
C23	−0.27629 (10)	0.5428 (2)	0.17870 (5)	0.0184 (3)	
H23	−0.2399	0.5296	0.2081	0.022*	
C24	−0.37414 (10)	0.5663 (2)	0.18048 (6)	0.0233 (3)	
H24	−0.4026	0.5730	0.2109	0.028*	
C25	−0.42886 (10)	0.5797 (2)	0.13671 (6)	0.0257 (4)	
H25	−0.4944	0.5943	0.1376	0.031*	
C26	−0.38589 (10)	0.5714 (2)	0.09141 (6)	0.0235 (3)	
H26	−0.4235	0.5781	0.0622	0.028*	
C27	−0.28759 (10)	0.5533 (2)	0.08876 (5)	0.0181 (3)	
C28	−0.24307 (11)	0.5548 (3)	0.03907 (5)	0.0240 (3)	
H28A	−0.2917	0.5402	0.0133	0.036*	
H28B	−0.1979	0.4620	0.0376	0.036*	
H28C	−0.2112	0.6633	0.0346	0.036*	
C51	0.26791 (9)	0.4681 (2)	0.18393 (5)	0.0144 (3)	
C52	0.28521 (10)	0.4299 (2)	0.23427 (5)	0.0195 (3)	
H52	0.2572	0.3323	0.2476	0.023*	
C53	0.34327 (11)	0.5341 (3)	0.26488 (6)	0.0245 (4)	
H53	0.3548	0.5050	0.2981	0.029*	
C54	0.38370 (11)	0.6815 (2)	0.24562 (6)	0.0242 (3)	
H54	0.4216	0.7533	0.2659	0.029*	
C55	0.36716 (10)	0.7212 (2)	0.19578 (6)	0.0215 (3)	
H55	0.3942	0.8206	0.1830	0.026*	
C56	0.31087 (9)	0.6156 (2)	0.16419 (5)	0.0162 (3)	
C57	0.30179 (10)	0.6591 (2)	0.10950 (5)	0.0214 (3)	
H57A	0.3332	0.7671	0.1037	0.032*	
H57B	0.2357	0.6686	0.0993	0.032*	
H57C	0.3307	0.5692	0.0908	0.032*	
C61	0.33606 (9)	0.1507 (2)	0.11991 (5)	0.0140 (3)	
C62	0.42569 (10)	0.1631 (2)	0.14398 (5)	0.0176 (3)	
H62	0.4307	0.1691	0.1784	0.021*	
C63	0.50781 (10)	0.1664 (2)	0.11694 (6)	0.0216 (3)	
H63	0.5672	0.1750	0.1334	0.026*	
C64	0.50103 (10)	0.1570 (2)	0.06563 (6)	0.0222 (3)	
H64	0.5558	0.1591	0.0475	0.027*	
C65	0.41180 (10)	0.1444 (2)	0.04118 (5)	0.0211 (3)	
H65	0.4069	0.1382	0.0067	0.025*	
C66	0.33007 (10)	0.1409 (2)	0.06832 (5)	0.0176 (3)	
H66	0.2708	0.1319	0.0518	0.021*	
N1	−0.01604 (8)	0.17311 (17)	0.20624 (4)	0.0136 (2)	
N2	0.16681 (8)	0.06105 (18)	0.12630 (4)	0.0148 (2)	
O1	0.06835 (7)	0.60208 (15)	0.13039 (4)	0.0166 (2)	
O2	0.03037 (7)	−0.09871 (14)	0.18344 (3)	0.0162 (2)	
H2	0.0298	−0.1727	0.1616	0.024*	

### Atomic displacement parameters (Å^2^)

**Table:**

	*U*^11^	*U*^22^	*U*^33^	*U*^12^	*U*^13^	*U*^23^
C1	0.0134 (5)	0.0153 (7)	0.0134 (6)	0.0008 (5)	0.0025 (4)	−0.0012 (5)
C2	0.0136 (5)	0.0135 (7)	0.0143 (6)	−0.0004 (5)	0.0016 (4)	−0.0029 (5)
C3	0.0138 (5)	0.0159 (7)	0.0092 (5)	−0.0001 (5)	0.0002 (4)	−0.0025 (5)
C4	0.0118 (5)	0.0132 (7)	0.0108 (5)	0.0000 (5)	0.0002 (4)	−0.0006 (5)
C5	0.0117 (5)	0.0135 (7)	0.0119 (5)	0.0002 (5)	0.0002 (4)	−0.0007 (5)
C6	0.0121 (5)	0.0158 (7)	0.0127 (5)	0.0008 (5)	−0.0003 (4)	0.0000 (5)
C7	0.0116 (5)	0.0122 (7)	0.0109 (5)	0.0004 (5)	0.0007 (4)	−0.0009 (5)
C8	0.0134 (5)	0.0130 (7)	0.0109 (5)	−0.0002 (5)	0.0014 (4)	0.0005 (5)
C9	0.0143 (5)	0.0166 (7)	0.0109 (5)	−0.0003 (5)	0.0005 (4)	−0.0016 (5)
C10	0.0137 (5)	0.0126 (7)	0.0142 (6)	0.0008 (5)	0.0003 (4)	−0.0016 (5)
C11	0.0148 (5)	0.0131 (7)	0.0121 (5)	0.0010 (5)	0.0004 (4)	−0.0024 (5)
C12	0.0146 (5)	0.0121 (7)	0.0115 (5)	0.0012 (5)	0.0006 (4)	−0.0021 (5)
C13	0.0171 (6)	0.0188 (8)	0.0143 (6)	−0.0002 (6)	0.0027 (5)	−0.0002 (6)
C14	0.0259 (7)	0.0240 (9)	0.0109 (6)	0.0033 (7)	0.0018 (5)	0.0017 (6)
C15	0.0239 (7)	0.0224 (8)	0.0122 (6)	0.0059 (6)	−0.0034 (5)	−0.0011 (6)
C16	0.0173 (6)	0.0162 (7)	0.0151 (6)	0.0025 (6)	−0.0021 (5)	−0.0039 (6)
C17	0.0159 (6)	0.0220 (9)	0.0218 (7)	0.0012 (6)	−0.0048 (5)	−0.0062 (6)
C18	0.0147 (6)	0.0216 (8)	0.0255 (7)	−0.0025 (6)	0.0006 (5)	−0.0049 (6)
C19	0.0169 (6)	0.0167 (7)	0.0175 (6)	−0.0023 (6)	0.0038 (5)	−0.0022 (6)
C21	0.0137 (6)	0.0158 (7)	0.0163 (6)	0.0006 (6)	0.0020 (4)	−0.0006 (6)
C22	0.0138 (6)	0.0144 (7)	0.0197 (6)	0.0007 (6)	0.0016 (5)	−0.0003 (6)
C23	0.0178 (6)	0.0180 (8)	0.0198 (6)	0.0013 (6)	0.0033 (5)	0.0000 (6)
C24	0.0186 (6)	0.0236 (9)	0.0282 (7)	0.0004 (6)	0.0080 (5)	−0.0007 (7)
C25	0.0138 (6)	0.0261 (9)	0.0373 (9)	0.0003 (6)	0.0031 (6)	0.0000 (7)
C26	0.0167 (6)	0.0239 (9)	0.0293 (8)	−0.0004 (6)	−0.0043 (5)	0.0018 (7)
C27	0.0163 (6)	0.0163 (8)	0.0215 (7)	0.0008 (6)	−0.0004 (5)	0.0009 (6)
C28	0.0237 (7)	0.0296 (10)	0.0187 (7)	0.0060 (7)	0.0003 (5)	0.0008 (7)
C51	0.0117 (5)	0.0157 (7)	0.0158 (6)	−0.0001 (5)	0.0009 (4)	−0.0035 (5)
C52	0.0183 (6)	0.0236 (8)	0.0165 (6)	−0.0053 (6)	0.0004 (5)	−0.0009 (6)
C53	0.0227 (7)	0.0336 (10)	0.0170 (6)	−0.0058 (7)	−0.0006 (5)	−0.0054 (7)
C54	0.0197 (6)	0.0270 (9)	0.0256 (7)	−0.0039 (7)	−0.0016 (5)	−0.0110 (7)
C55	0.0174 (6)	0.0176 (8)	0.0296 (8)	−0.0025 (6)	0.0007 (5)	−0.0030 (7)
C56	0.0127 (5)	0.0156 (7)	0.0203 (6)	0.0012 (6)	0.0015 (5)	−0.0012 (6)
C57	0.0201 (6)	0.0212 (8)	0.0227 (7)	−0.0030 (6)	−0.0003 (5)	0.0056 (6)
C61	0.0123 (5)	0.0129 (7)	0.0167 (6)	0.0017 (5)	0.0001 (4)	0.0000 (5)
C62	0.0157 (6)	0.0200 (8)	0.0169 (6)	0.0008 (6)	−0.0013 (5)	0.0001 (6)
C63	0.0126 (6)	0.0255 (9)	0.0265 (7)	0.0003 (6)	−0.0011 (5)	−0.0006 (7)
C64	0.0159 (6)	0.0252 (9)	0.0260 (7)	0.0004 (6)	0.0064 (5)	−0.0039 (7)
C65	0.0205 (6)	0.0260 (9)	0.0172 (6)	0.0006 (7)	0.0038 (5)	−0.0035 (6)
C66	0.0148 (6)	0.0221 (8)	0.0158 (6)	0.0006 (6)	−0.0002 (5)	−0.0023 (6)
N1	0.0140 (5)	0.0158 (6)	0.0109 (5)	−0.0001 (5)	0.0009 (4)	−0.0016 (5)
N2	0.0119 (5)	0.0143 (6)	0.0183 (5)	0.0006 (5)	0.0005 (4)	−0.0024 (5)
O1	0.0160 (4)	0.0152 (5)	0.0188 (5)	0.0001 (4)	0.0024 (3)	0.0014 (4)
O2	0.0228 (5)	0.0121 (5)	0.0137 (4)	0.0018 (4)	0.0008 (4)	0.0007 (4)

### Geometric parameters (Å, º)

**Table:**

C1—N1	1.4750 (18)	C21—C22	1.4720 (18)
C1—C2	1.524 (2)	C21—H21	0.9300
C1—H1A	0.9700	C22—C23	1.3997 (19)
C1—H1B	0.9700	C22—C27	1.4113 (19)
C2—C21	1.3451 (19)	C23—C24	1.3916 (19)
C2—C3	1.4956 (17)	C23—H23	0.9300
C3—O1	1.2303 (18)	C24—C25	1.384 (2)
C3—C4	1.510 (2)	C24—H24	0.9300
C4—C5	1.5343 (17)	C25—C26	1.389 (2)
C4—C9	1.5598 (18)	C25—H25	0.9300
C4—C7	1.565 (2)	C26—C27	1.3950 (19)
C5—C51	1.5146 (19)	C26—H26	0.9300
C5—C6	1.546 (2)	C27—C28	1.505 (2)
C5—H5	0.9800	C28—H28A	0.9600
C6—N2	1.4757 (18)	C28—H28B	0.9600
C6—C61	1.5173 (18)	C28—H28C	0.9600
C6—H6	0.9800	C51—C52	1.3997 (19)
C7—N2	1.4730 (17)	C51—C56	1.405 (2)
C7—C12	1.5192 (18)	C52—C53	1.391 (2)
C7—C8	1.6082 (18)	C52—H52	0.9300
C8—O2	1.4131 (17)	C53—C54	1.384 (3)
C8—N1	1.4747 (17)	C53—H53	0.9300
C8—C10	1.5133 (18)	C54—C55	1.388 (2)
C9—N1	1.4718 (17)	C54—H54	0.9300
C9—H9A	0.9700	C55—C56	1.399 (2)
C9—H9B	0.9700	C55—H55	0.9300
C10—C19	1.3721 (18)	C56—C57	1.512 (2)
C10—C11	1.4073 (18)	C57—H57A	0.9600
C11—C12	1.4082 (18)	C57—H57B	0.9600
C11—C16	1.4083 (18)	C57—H57C	0.9600
C12—C13	1.3703 (18)	C61—C66	1.3921 (18)
C13—C14	1.4241 (19)	C61—C62	1.3951 (18)
C13—H13	0.9300	C62—C63	1.3940 (19)
C14—C15	1.372 (2)	C62—H62	0.9300
C14—H14	0.9300	C63—C64	1.385 (2)
C15—C16	1.417 (2)	C63—H63	0.9300
C15—H15	0.9300	C64—C65	1.395 (2)
C16—C17	1.421 (2)	C64—H64	0.9300
C17—C18	1.380 (2)	C65—C66	1.3910 (19)
C17—H17	0.9300	C65—H65	0.9300
C18—C19	1.416 (2)	C66—H66	0.9300
C18—H18	0.9300	N2—H2A	0.93 (2)
C19—H19	0.9300	O2—H2	0.8200
			
N1—C1—C2	114.20 (10)	C18—C19—H19	120.7
N1—C1—H1A	108.7	C2—C21—C22	128.81 (13)
C2—C1—H1A	108.7	C2—C21—H21	115.6
N1—C1—H1B	108.7	C22—C21—H21	115.6
C2—C1—H1B	108.7	C23—C22—C27	119.23 (13)
H1A—C1—H1B	107.6	C23—C22—C21	121.02 (13)
C21—C2—C3	116.37 (12)	C27—C22—C21	119.51 (12)
C21—C2—C1	125.87 (12)	C24—C23—C22	121.09 (14)
C3—C2—C1	117.52 (12)	C24—C23—H23	119.5
O1—C3—C2	122.21 (13)	C22—C23—H23	119.5
O1—C3—C4	121.78 (12)	C25—C24—C23	119.56 (14)
C2—C3—C4	116.00 (12)	C25—C24—H24	120.2
C3—C4—C5	114.87 (12)	C23—C24—H24	120.2
C3—C4—C9	106.86 (10)	C24—C25—C26	119.97 (14)
C5—C4—C9	119.19 (10)	C24—C25—H25	120.0
C3—C4—C7	111.75 (10)	C26—C25—H25	120.0
C5—C4—C7	102.88 (10)	C25—C26—C27	121.44 (14)
C9—C4—C7	100.20 (11)	C25—C26—H26	119.3
C51—C5—C4	119.13 (11)	C27—C26—H26	119.3
C51—C5—C6	114.20 (11)	C26—C27—C22	118.67 (13)
C4—C5—C6	102.49 (11)	C26—C27—C28	119.89 (13)
C51—C5—H5	106.7	C22—C27—C28	121.42 (12)
C4—C5—H5	106.7	C27—C28—H28A	109.5
C6—C5—H5	106.7	C27—C28—H28B	109.5
N2—C6—C61	112.81 (11)	H28A—C28—H28B	109.5
N2—C6—C5	103.45 (10)	C27—C28—H28C	109.5
C61—C6—C5	111.10 (12)	H28A—C28—H28C	109.5
N2—C6—H6	109.8	H28B—C28—H28C	109.5
C61—C6—H6	109.8	C52—C51—C56	118.67 (13)
C5—C6—H6	109.8	C52—C51—C5	120.07 (13)
N2—C7—C12	111.75 (10)	C56—C51—C5	121.18 (12)
N2—C7—C4	104.53 (10)	C53—C52—C51	121.65 (15)
C12—C7—C4	117.16 (12)	C53—C52—H52	119.2
N2—C7—C8	116.49 (12)	C51—C52—H52	119.2
C12—C7—C8	103.71 (10)	C54—C53—C52	119.58 (15)
C4—C7—C8	103.41 (10)	C54—C53—H53	120.2
O2—C8—N1	104.98 (10)	C52—C53—H53	120.2
O2—C8—C10	113.56 (12)	C53—C54—C55	119.42 (15)
N1—C8—C10	115.24 (11)	C53—C54—H54	120.3
O2—C8—C7	112.30 (10)	C55—C54—H54	120.3
N1—C8—C7	105.94 (11)	C54—C55—C56	121.76 (16)
C10—C8—C7	104.74 (10)	C54—C55—H55	119.1
N1—C9—C4	103.36 (10)	C56—C55—H55	119.1
N1—C9—H9A	111.1	C55—C56—C51	118.88 (13)
C4—C9—H9A	111.1	C55—C56—C57	118.99 (14)
N1—C9—H9B	111.1	C51—C56—C57	122.07 (13)
C4—C9—H9B	111.1	C56—C57—H57A	109.5
H9A—C9—H9B	109.1	C56—C57—H57B	109.5
C19—C10—C11	119.56 (12)	H57A—C57—H57B	109.5
C19—C10—C8	131.59 (12)	C56—C57—H57C	109.5
C11—C10—C8	108.84 (11)	H57A—C57—H57C	109.5
C10—C11—C12	113.44 (12)	H57B—C57—H57C	109.5
C10—C11—C16	123.08 (12)	C66—C61—C62	118.77 (12)
C12—C11—C16	123.47 (13)	C66—C61—C6	120.88 (12)
C13—C12—C11	118.88 (12)	C62—C61—C6	120.24 (12)
C13—C12—C7	131.88 (12)	C63—C62—C61	120.72 (13)
C11—C12—C7	109.24 (11)	C63—C62—H62	119.6
C12—C13—C14	118.67 (13)	C61—C62—H62	119.6
C12—C13—H13	120.7	C64—C63—C62	120.04 (13)
C14—C13—H13	120.7	C64—C63—H63	120.0
C15—C14—C13	122.37 (13)	C62—C63—H63	120.0
C15—C14—H14	118.8	C63—C64—C65	119.74 (13)
C13—C14—H14	118.8	C63—C64—H64	120.1
C14—C15—C16	120.16 (13)	C65—C64—H64	120.1
C14—C15—H15	119.9	C66—C65—C64	120.01 (13)
C16—C15—H15	119.9	C66—C65—H65	120.0
C11—C16—C15	116.39 (13)	C64—C65—H65	120.0
C11—C16—C17	116.23 (13)	C65—C66—C61	120.73 (13)
C15—C16—C17	127.38 (13)	C65—C66—H66	119.6
C18—C17—C16	120.37 (13)	C61—C66—H66	119.6
C18—C17—H17	119.8	C9—N1—C8	103.62 (10)
C16—C17—H17	119.8	C9—N1—C1	108.17 (12)
C17—C18—C19	122.17 (13)	C8—N1—C1	116.07 (10)
C17—C18—H18	118.9	C7—N2—C6	111.07 (11)
C19—C18—H18	118.9	C7—N2—H2A	108.8 (11)
C10—C19—C18	118.53 (13)	C6—N2—H2A	111.1 (11)
C10—C19—H19	120.7	C8—O2—H2	109.5
			
N1—C1—C2—C21	149.63 (14)	C12—C11—C16—C15	2.2 (2)
N1—C1—C2—C3	−24.42 (17)	C10—C11—C16—C17	1.9 (2)
C21—C2—C3—O1	26.7 (2)	C12—C11—C16—C17	−177.20 (14)
C1—C2—C3—O1	−158.71 (13)	C14—C15—C16—C11	−2.5 (2)
C21—C2—C3—C4	−152.30 (13)	C14—C15—C16—C17	176.78 (16)
C1—C2—C3—C4	22.32 (17)	C11—C16—C17—C18	−1.6 (2)
O1—C3—C4—C5	1.50 (18)	C15—C16—C17—C18	179.11 (16)
C2—C3—C4—C5	−179.52 (11)	C16—C17—C18—C19	−0.3 (3)
O1—C3—C4—C9	136.12 (13)	C11—C10—C19—C18	−1.5 (2)
C2—C3—C4—C9	−44.91 (15)	C8—C10—C19—C18	177.39 (15)
O1—C3—C4—C7	−115.20 (14)	C17—C18—C19—C10	1.9 (2)
C2—C3—C4—C7	63.77 (14)	C3—C2—C21—C22	179.94 (14)
C3—C4—C5—C51	72.82 (15)	C1—C2—C21—C22	5.8 (3)
C9—C4—C5—C51	−55.89 (19)	C2—C21—C22—C23	41.3 (2)
C7—C4—C5—C51	−165.51 (12)	C2—C21—C22—C27	−144.41 (17)
C3—C4—C5—C6	−160.01 (11)	C27—C22—C23—C24	−1.9 (2)
C9—C4—C5—C6	71.28 (15)	C21—C22—C23—C24	172.40 (15)
C7—C4—C5—C6	−38.34 (12)	C22—C23—C24—C25	2.3 (3)
C51—C5—C6—N2	166.72 (10)	C23—C24—C25—C26	−0.7 (3)
C4—C5—C6—N2	36.46 (12)	C24—C25—C26—C27	−1.4 (3)
C51—C5—C6—C61	−71.98 (14)	C25—C26—C27—C22	1.7 (3)
C4—C5—C6—C61	157.77 (10)	C25—C26—C27—C28	−176.57 (17)
C3—C4—C7—N2	149.97 (11)	C23—C22—C27—C26	−0.1 (2)
C5—C4—C7—N2	26.21 (13)	C21—C22—C27—C26	−174.49 (15)
C9—C4—C7—N2	−97.13 (11)	C23—C22—C27—C28	178.19 (16)
C3—C4—C7—C12	25.69 (15)	C21—C22—C27—C28	3.8 (2)
C5—C4—C7—C12	−98.08 (12)	C4—C5—C51—C52	63.48 (18)
C9—C4—C7—C12	138.59 (11)	C6—C5—C51—C52	−57.98 (16)
C3—C4—C7—C8	−87.65 (12)	C4—C5—C51—C56	−119.80 (14)
C5—C4—C7—C8	148.58 (10)	C6—C5—C51—C56	118.73 (14)
C9—C4—C7—C8	25.25 (12)	C56—C51—C52—C53	0.2 (2)
N2—C7—C8—O2	1.91 (16)	C5—C51—C52—C53	177.00 (14)
C12—C7—C8—O2	125.13 (11)	C51—C52—C53—C54	1.3 (2)
C4—C7—C8—O2	−112.11 (12)	C52—C53—C54—C55	−1.2 (2)
N2—C7—C8—N1	115.97 (12)	C53—C54—C55—C56	−0.3 (2)
C12—C7—C8—N1	−120.81 (11)	C54—C55—C56—C51	1.8 (2)
C4—C7—C8—N1	1.96 (13)	C54—C55—C56—C57	−175.45 (14)
N2—C7—C8—C10	−121.77 (12)	C52—C51—C56—C55	−1.7 (2)
C12—C7—C8—C10	1.45 (14)	C5—C51—C56—C55	−178.49 (13)
C4—C7—C8—C10	124.21 (11)	C52—C51—C56—C57	175.45 (13)
C3—C4—C9—N1	71.24 (13)	C5—C51—C56—C57	−1.3 (2)
C5—C4—C9—N1	−156.48 (12)	N2—C6—C61—C66	33.4 (2)
C7—C4—C9—N1	−45.38 (13)	C5—C6—C61—C66	−82.26 (17)
O2—C8—C10—C19	57.4 (2)	N2—C6—C61—C62	−150.62 (14)
N1—C8—C10—C19	−63.8 (2)	C5—C6—C61—C62	93.74 (16)
C7—C8—C10—C19	−179.77 (16)	C66—C61—C62—C63	0.3 (2)
O2—C8—C10—C11	−123.63 (13)	C6—C61—C62—C63	−175.75 (15)
N1—C8—C10—C11	115.22 (13)	C61—C62—C63—C64	−0.2 (3)
C7—C8—C10—C11	−0.75 (15)	C62—C63—C64—C65	0.1 (3)
C19—C10—C11—C12	178.83 (14)	C63—C64—C65—C66	−0.1 (3)
C8—C10—C11—C12	−0.32 (17)	C64—C65—C66—C61	0.3 (3)
C19—C10—C11—C16	−0.4 (2)	C62—C61—C66—C65	−0.4 (2)
C8—C10—C11—C16	−179.54 (13)	C6—C61—C66—C65	175.66 (15)
C10—C11—C12—C13	−179.37 (14)	C4—C9—N1—C8	48.13 (13)
C16—C11—C12—C13	−0.2 (2)	C4—C9—N1—C1	−75.60 (12)
C10—C11—C12—C7	1.34 (17)	O2—C8—N1—C9	88.49 (12)
C16—C11—C12—C7	−179.45 (13)	C10—C8—N1—C9	−145.81 (12)
N2—C7—C12—C13	−54.6 (2)	C7—C8—N1—C9	−30.52 (13)
C4—C7—C12—C13	66.0 (2)	O2—C8—N1—C1	−153.10 (11)
C8—C7—C12—C13	179.16 (16)	C10—C8—N1—C1	−27.41 (16)
N2—C7—C12—C11	124.59 (13)	C7—C8—N1—C1	87.88 (13)
C4—C7—C12—C11	−114.86 (13)	C2—C1—N1—C9	52.05 (14)
C8—C7—C12—C11	−1.68 (15)	C2—C1—N1—C8	−63.82 (15)
C11—C12—C13—C14	−1.5 (2)	C12—C7—N2—C6	124.29 (12)
C7—C12—C13—C14	177.57 (15)	C4—C7—N2—C6	−3.38 (14)
C12—C13—C14—C15	1.2 (2)	C8—C7—N2—C6	−116.76 (13)
C13—C14—C15—C16	1.0 (3)	C61—C6—N2—C7	−140.82 (12)
C10—C11—C16—C15	−178.67 (14)	C5—C6—N2—C7	−20.68 (14)

### Hydrogen-bond geometry (Å, º)

**Table:**

*D*—H···*A*	*D*—H	H···*A*	*D*···*A*	*D*—H···*A*
O2—H2···O1^i^	0.82	2.02	2.7828 (15)	155
C1—H1*A*···O2^ii^	0.97	2.46	3.3040 (16)	145
C57—H57*B*···O1	0.96	2.59	3.3859 (18)	141
N2—H2*A*···O2	0.92 (2)	2.27 (2)	2.8016 (18)	117 (2)

Symmetry codes: (i) *x*, *y*−1, *z*; (ii) −*x*, *y*+1/2, −*z*+1/2.
